# Dental caries detection using a semi-supervised learning approach

**DOI:** 10.1038/s41598-023-27808-9

**Published:** 2023-01-13

**Authors:** Adnan Qayyum, Ahsen Tahir, Muhammad Atif Butt, Alexander Luke, Hasan Tahir Abbas, Junaid Qadir, Kamran Arshad, Khaled Assaleh, Muhammad Ali Imran, Qammer H. Abbasi

**Affiliations:** 1 James Watt School of Engineering, University of Glasgow, Glasgow, UK; 2 Information Technology University of the Punjab, Lahore, Pakistan; 3 Department of Electrical Engineering, University of Engineering and Technology, Lahore, Pakistan; 4 Department of Clinical Sciences, College of Dentistry, Ajman University, Ajman, UAE; 5 Centre of Medical and Bio-allied Health Sciences Research, Ajman University, Ajman, UAE; 6 Department of Computer Science and Engineering, College of Engineering, Qatar University, Doha, Qatar; 7 Artificial Intelligence Research Center (AIRC), College of Engineering and Information Technology, Ajman University, Ajman, UAE

**Keywords:** Biomedical engineering, Dentistry, Diagnosis, Medical imaging

## Abstract

Early diagnosis of dental caries progression can prevent invasive treatment and enable preventive treatment. In this regard, dental radiography is a widely used tool to capture dental visuals that are used for the detection and diagnosis of caries. Different deep learning (DL) techniques have been used to automatically analyse dental images for caries detection. However, most of these techniques require large-scale annotated data to train DL models. On the other hand, in clinical settings, such medical images are scarcely available and annotations are costly and time-consuming. To this end, we present an efficient self-training-based method for caries detection and segmentation that leverages a small set of labelled images for training the teacher model and a large collection of unlabelled images for training the student model. We also propose to use centroid cropped images of the caries region and different augmentation techniques for the training of self-supervised models that provide computational and performance gains as compared to fully supervised learning and standard self-supervised learning methods. We present a fully labelled dental radiographic dataset of 141 images that are used for the evaluation of baseline and proposed models. Our proposed self-supervised learning strategy has provided performance improvement of approximately 6% and 3% in terms of average pixel accuracy and mean intersection over union, respectively as compared to standard self-supervised learning. Data and code will be made available to facilitate future research.

## Introduction

Dental caries, also sometimes referred to as dental cavities or tooth decay, is one of the most prevalent global chronic diseases. The American Dental Association has classified dental caries into different grades by considering the spread and extent of lesions that include normal, initial, moderate, and extensive spread^1^. In clinical practice, diagnosing the initial posterior proximal caries using routine clinical examinations is very difficult^2^. To overcome these limitations, dental radiography is used as a major tool for the identification of dental caries that provides a visual depiction of the bitewing. Although dental radiography makes it easy for human experts to identify dental caries and other abnormalities, however, the detection of posterior initial proximal caries is quite challenging. However, working out a viable solution for this challenge can prevent invasive treatments and more importantly reduce healthcare costs.

Dental radiography despite being the most recommended and widely used tool for caries identification in dental practice is very subjective. The observations of different human experts (i.e., oral radiologists) vary and often contain major disparities in the diagnosis of initial caries (i.e., whether they are present or not). There are many factors influencing this subjectivity, such as radiographic image quality, expert expectations, viewing conditions, time consumed per examination, and variability across examiners^3^. In the literature, such a phenomenon has already been observed experimentally; for instance, 34 dentists showed notable disparities in analysing dental radiographs for caries identification^2^. Therefore, the development of automated tools for caries detection is required that will not only reduce the subjective bias associated with human examiners but will also enable early detection of initial caries (which are often overlooked). Such a method will also reduce the burden on oral radiologists who have to manually analyse large sets of images in their daily routine clinical practice.

In the literature, different deep learning (DL)-based solutions have been proposed for the identification of dental caries. However, one of the major limitations of these methods is that they require a large-scale annotated dataset for the training of DL models^4^. Whereas, in realistic clinical settings, such data collections are scarcely available^5^. Also, the annotation of unlabelled images is very costly, time-consuming, and unfeasible sometimes, e.g., due to the unavailability of human experts^6^. If we somehow manage to arrange for human experts to perform data labelling, it can be frustrating for expert radiologists to spend their valuable time fully understanding different annotation tools used by technical data annotators. This motivates the development of unsupervised methods that do not require large-scale labelled training data.

In addition to the data availability issue, most DL models need sufficiently high computational resources for their training, e.g., graphical processing units (GPUs) and tensor processing units (TPUs)^7,8^. On the contrary, such resources are generally not available in clinical settings. To overcome the aforementioned challenges, we present a low-cost self-supervised learning-based framework for the development of an efficient caries detection model in dental radiographs. The following are the major contributions of this paper.

1. We present the dental caries detection dataset (DCD$^{2}$) containing 229 dental X-ray images for the caries detection problem that contains 141 annotated and 88 unlabelled images. Alongside, we also present benchmarks by evaluating state-of-the-art DL segmentation models in a supervised learning setting using real labelled data.
2. We present a student-teacher method-based self-training framework for caries detection that leverages both labelled and unlabelled images. To improve self-training, we propose a centroid cropping-based sampling (CCS) method for extracting caries region(s) in dental X-ray images for the development of low-cost and efficient self-supervised learning.
3. We perform an extensive experimental evaluation of the proposed method using DCD$^{2}$) that includes validating the performance of various teacher and student models using varying input samples and the same model architecture used in teacher and student networks. We also evaluate the generalisability of self-training across different architectures of the student model.

## Related work

In literature, different methods for caries detection have been presented including traditional image processing-based methods and as well as DL-based methods. For instance, Geetha et al.^9^ utilised statistical features obtained from Laplacian/Gaussian filters and image dilation and erosion operations for classification with MLP to detect caries. Prerna et al.^10^ first applied a median filter for noise removal in dental radiographs and then trained CNN and LSTM-based hybrid models for caries segmentation. Similarly, the use of different image processing operations such as Gaussian filtering and Sobel operator for caries segmentation in intra-oral radiographs is presented in^11^. A Principal Component Analysis (PCA) was then performed on the obtained features for dimensionality reduction, and a Multi-Layer Perceptron (MLP) was trained for the detection of caries to provide a detection accuracy of 89% on the dental radiographs. Rad et al.^12^ utilised an MLP neural network model for the classification of caries. Moreover, the authors extracted teeth from images using segmentation and applied the model both to the images and the extracted segments with an accuracy performance of 90% and 98%, respectively.

Moutselos et al.^13^ applied a Mask R-CNN model for caries recognition. They applied various image augmentation operations that include flipping, rotations, and affine transformations to increase the training data for efficient learning of the underlying model. Vinayahalingam et al.^14^ proposed using the MobileNet V2 model for the detection of caries in mandibular as well as maxillary molars. Muthu et al.^15^ first extracted features from panoramic radiographs and then trained the AlexNet model for the detection of caries which was formulated as a classification problem. Vinayahalingam et al.^16^ performed manual extraction of regions of interest (ROI) in the image and applied the MobileNetV2 model for caries classification. Haghanifar et al.^17^ performed various preprocessing steps, including vertical edge filtering, Gaussian and bilateral filtering, along with Savoula binarization, before extraction of ROI and features from the radiographs, which are then used as input to the proposed DL-based model named PaXNet. Cantu et al.^18^ utilised image augmentation operations such as flipping, cropping, translations, and rotations before applying sharpening and contrast operations for classification by a U-Net segmentation model for the detection of caries. Similarly, Ezhov et al.^19^ used tooth localization and ROI extraction before performing image segmentation for caries with a U-Net model. Zhang et al.^20^ utilised a single-shot detector DL model for the detection of caries from intra-oral photographs. Javid et al.^21^ sharpened the dental images with a sharpening filter before applying a Mask Region-based CNN (R-CNN) for the detection of caries.

Khan et al.^22^ proposed a combination of DL models that include U-Net and DenseNet121 for caries detection. The authors performed image augmentation steps of flipping and rotation before using the images for the training of models. Similarly, Casalegno et al^23^, performed rotation, translation, and contrast transformations to the image before using the augmented images as input to the U-Net and VGG16 models for caries segmentation. Jung et al.^24^ presented an autoencoder-based model, DeepLab-v3, which is based on the ResNet18 model, for multi-classification into 6 classes, including caries. In contrast to the aforementioned articles that mainly rely on labelled training data, we present a self-training-based semi-supervised learning approach that only utilises 20 labelled images for the training of a teacher model. Then the trained teacher model is inferred to get pseudo labels for unlabelled images that are used to train the student model in a self-supervised learning fashion. To the best of our knowledge, this paper is the first attempt towards leveraging self-supervised learning for dental caries segmentation.

## Methodology

**Figure 1:**
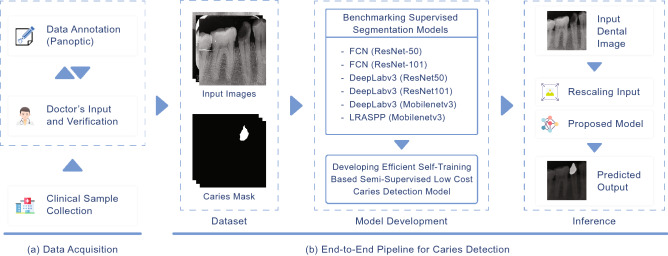
Illustration of our proposed method for caries detection in dental X-ray images that consists of two major parts (1) data collection and annotation; and (2) end-to-end training of caries detection models.

In this section, we present our proposed methodology for caries detection in dental radiographs, which is mainly illustrated in Fig. 1. We will start this section by first describing the data collection process and formally formulating the problem.

### Dental Caries Detection Dataset (DCD$^{2}$)

#### Data collection strategy

The data collection process involves two main steps (as depicted in Fig. 1), i.e., clinical sample collection and panoptic annotation and verification by an expert dentist. The data collection process was carried out in the College of Medicine, Ajman University, United Arab Emirates and a MyRay X-ray scanner was used for data collection. Note that informed consent from data subjects and ethical approval (having reference number: D-H-F April 25) from Research Ethics Committee, Ajman University, UAE was obtained before initiating the data collection and all ethical guidelines were followed in the data collection, annotation, and analysis processes.

#### Data preprocessing and annotation

The annotation of dental caries requires pixel-level identification of the caries region and to accomplish this task we carefully designed a data annotation method that comprises three steps: (1) training of a data annotators team by a dental expert; (2) annotation of dental images by carefully following the guidelines provided by the expert; and (3) validation and rectification of annotations by expert. The oral radiologist has more than 20 years of field experience and we considered only those annotations that were verified by him. We used a widely used tool named “Labelme” for annotating dental radiographs^25^. Moreover, appropriate preprocessing was applied to all images to eliminate any privacy-related information. As it is very common in radiography to have patients’ names on the X-ray image, such images were cropped to ensure the privacy of patients.

#### Data statistics

**Figure 2:**
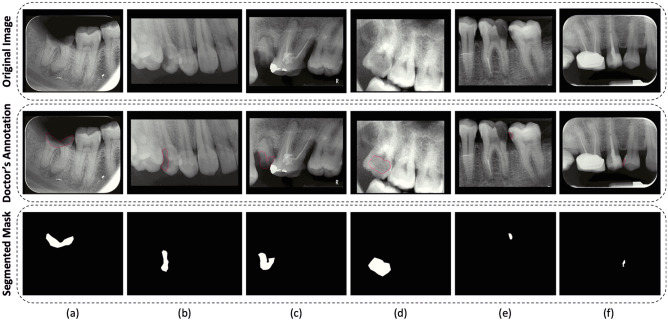
Illustration of different variations in our dataset. The first, second, and third rows contain original images, the doctor’s annotation, and the corresponding generated mask, respectively.

The final dataset contains a total of 229 dental radiographs of which 141 are annotated and 88 are unlabelled. In our dataset, there are a total of 114 male dental scans and 115 female dental scans. The labelled images were used for the evaluation of DL models trained using fully supervised learning and self-supervised learning strategies. A visual depiction of different data variations along with a generated segmentation mask (using doctor’s annotations) for the training of DL models is presented in Fig. 2.

### Problem formulation

We have formulated caries detection as a segmentation problem in which we are interested in segmenting a dental X-ray image into two components, i.e., background (region without caries) and foreground (region containing caries). As discussed above, in medical settings, data annotation is very challenging due to the annotation cost, time, and availability of human experts, e.g., physicians and radiologists. Considering such a case, we have formulated caries detection as a self-supervised learning problem. Let’s assume we have two sets of data samples, i.e., annotated and unannotated images. We denote the labelled dataset as ${\mathscr {D}}_L = \{(x_i^l,y_i^l)\}_{i=1}^{N_l}$, which is used to train the teacher model ${\mathscr {M}}_T$ in supervised learning fashion. Where, $x_i^l$ and $y_i^l$ represent the labelled dental X-ray image and its corresponding label, respectively. $N_l$ denotes the total number of labelled images and $y_i^l \in \{0,1\}$ denotes labelled binary images consisting of 0 and 1 representing background and foreground (caries region in our case), respectively. Unlabelled dataset is denoted as ${\mathscr {D}}_U = \{(x_j^u)\}_{j=1}^{N_u}$, which is used to train the student model ${\mathscr {M}}_S$, where $N_u$ is the total number of labelled images. To train ${\mathscr {M}}_S$ using self-training method, we first get the pseudo label of unlabelled input $x_j^u$ (which is denoted as $y_j^p$), then the pair $\{x_j^u,y_j^p\}$ is used to create a pseudo label dataset ${\mathscr {D}}_P = \{(x_j^u,y_j^p)\}$, which is used for training student model. In this way, binary cross entropy loss (as given in Eq. 1) is minimized to enhance the performance of student model ${\mathscr {M}}_S$ in segmenting caries region in unlabelled dental radiographic images (i.e., $x_j^u$).

1 $$\begin{aligned} {\mathscr {L}}(y_j^p,\hat{y}_j) = y_j^p \log (\hat{y}_j - (1-y_j^p) \log (1-\hat{y}_j)) \end{aligned}$$

Where, $\hat{y}_j$ represents the predicted mask from the neural network (i.e., the output of the student segmentation model) and $y_j^p$ is the pseudo label generated by the teacher model. Our proposed method for efficient self-supervised learning is described next.

### Proposed self-training method for caries segmentation

**Figure 3:**
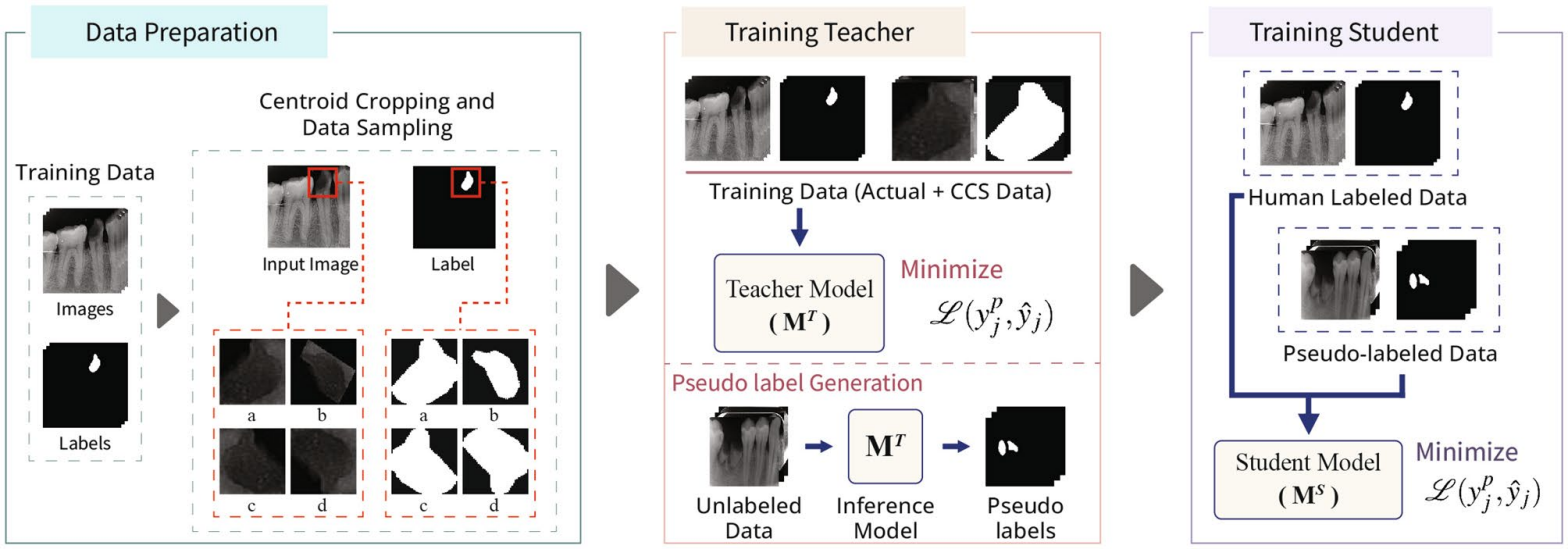
An overview of our proposed self-supervised learning-based method for dental caries segmentation. Firstly, the training data is re-sampled through our centroid cropping-based sampling (CCS) approach that initially extracts the cavity region from the input images and employs state-of-the-art transformation techniques to increase the data samples. Secondly, the teacher model ${\mathscr {M}}_T$ is trained in a fully supervised learning fashion on real data (to guarantee high-quality pseudo-label generation), which is then used to generate pseudo labels for unlabelled images for training student model ${\mathscr {M}}_S$. Lastly, the student model is trained on both the real and pseudo labels to ensure better generalization.

**Figure 4:**
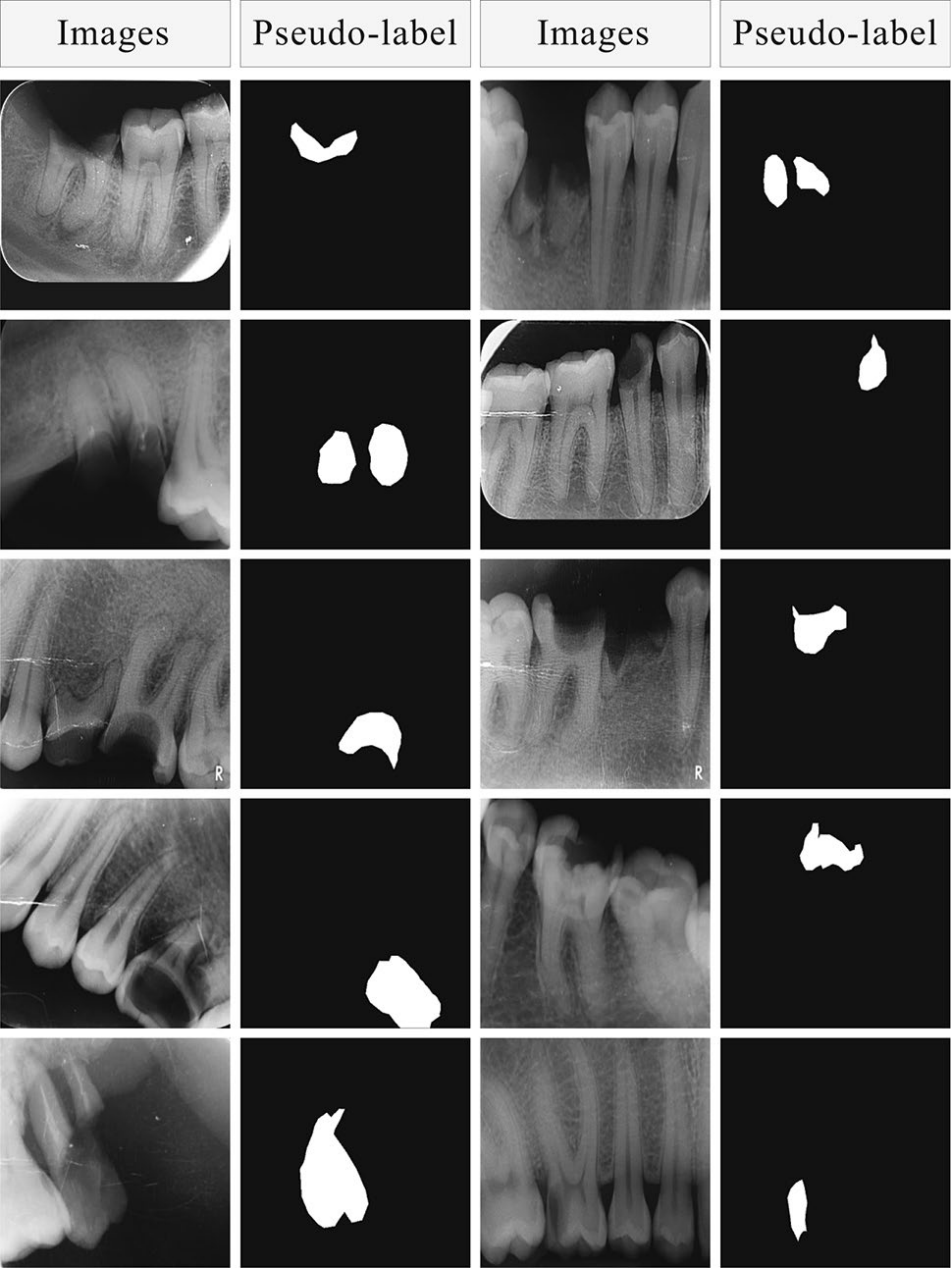
Visual examples of pseudo label generation using teacher model ${\mathscr {M}}_T$ that are used for training student model ${\mathscr {M}}_S$ in conjunction with unlabelled data ${\mathscr {D}}_U$. It can be seen that ${\mathscr {M}}_T$ has accurately predicted the pseudo labels for unlabelled images (it is also supported by the quantitative results).

Our proposed efficient self-training method for caries image segmentation is depicted in Fig. 3. Our method contains two models, i.e., the teacher model (${\mathscr {M}}_T$) and the student model (${\mathscr {M}}_S$). Initially, ${\mathscr {M}}_T$ is trained using a small set of labelled images ${\mathscr {D}}_L$ (we evaluated different number of images for training ${\mathscr {M}}_T$ in a fully supervised learning strategy). Then unlabelled images (i.e., ${\mathscr {D}}_U = \{(x_j^u)\}_{j=1}^{N_u}$) are used to infer ${\mathscr {M}}_T$ to get pseudo labels (i.e., $y_j^p$) for unlabelled images, which are then merged with the corresponding unlabelled images to form a pair $\{x_{j_u},y_j^p\}$ that is used for training student model ${\mathscr {M}}_S$ in supervised learning fashion. The generated pseudo labels for five examples of unlabelled images using trained ${\mathscr {M}}_T$ are demonstrated in Fig. 4. The figure highlights that the trained teacher model was efficiently able to capture the problem-specific features (i.e., caries information) from unlabelled dental X-ray images, which was used for training ${\mathscr {M}}_S$ in the self-supervised learning paradigm. We also proposed to dynamically crop the caries region from the dental X-ray to significantly reduce the size of input image pairs used for training our DL models in self-supervised learning, i.e., centroid cropping-based sampling (CCS). This strategy improves the overall training process of underlying DL models in terms of training time and also results in the development of a low-cost solution for caries segmentation. Note that initially, we train the ${\mathscr {M}}_S$ as a single pair (i.e., $\{x_{j_u},y_j^p\}$) to get the baseline results for standard self-supervised learning. Then to improve the performance of ${\mathscr {M}}_S$, we used different data augmentation techniques namely horizontal flip, shear, rotation, and vertical flip that were applied on the cropped image pair (i.e., $\{x_{j_c}^u,y_{j_c}^p\}$). An illustration of these augmentation techniques, when applied to the cropped patches of input images containing caries and their corresponding, cropped segmentation masks is shown in the first block of Fig. 3. Applying these augmentations consequently increased the size of the training set used for optimizing ${\mathscr {M}}_S$, i.e., after performing these data augmentation techniques, we have 635 images in the training set. Therefore, this technique provided significant performance improvement in terms of different performance metrics. The results of baseline models and our proposed framework are described in the next section.

### Benchmarking fully supervised baseline models for caries segmentation

As one of the prime contributions of this paper is a fully labelled database of 141 images containing dental radiographic scans. Therefore, we first evaluated six different state-of-the-art models for caries detection that consists of two parts, i.e., the generative model and the backbone classification model. The key role of the backbone network is to learn the pixel-wise binary classification foreground (caries region) and background by optimizing binary cross entropy loss (defined in Eq. 1). Note that for model training using fully supervised learning strategy, the loss is computed between the predicted mask by the model and the ground truth (actual human labelled) mask. Specifically, we have used three generative models and three detector networks that are used for the generation of caries segmentation masks (prediction of the models). The generative models are used for the generating final segmentation mask that includes Deeplab-v3^26^, fully convolutional network (FCN)^27^, and Lite Reduced Atrous Spatial Pyramid Pooling (LRASPP)^28^. In addition, these models work with a classifier model working as the backbone that learns pixel-level classification of caries region and background region. The classifier models that are used as a backbone of segmentation models include ResNet-50^29^, ResNet-101^29^, and Mobilenet-v3^30^. All these models are state-of-the-art models used in benchmarking segmentation datasets. Benchmark results using the baseline supervised learning models will be presented in the next section.

## Experiments and results

In this section, we present the results of our proposed efficient self-supervised learning framework for caries segmentation. Results of our proposed method are also compared with two baseline approaches that include models trained in fully supervised settings and models trained using a standard self-supervised learning strategy. We will first briefly discuss the data description and experimental setup that was used for training our proposed framework and baseline approaches.

### Data description and experimental setup

As discussed above, our dataset contains a total of 141 labelled images. Firstly, to get the baseline results, we train six models in supervised learning settings using a split of 90% and 10% for training and testing sets, respectively. For semi-supervised training methods, we randomly selected different number of images from the training set for training of teacher model ${\mathscr {M}}_T$ and the remaining images were considered as unlabelled images that were used for the generation of pseudo labels (from trained ${\mathscr {M}}_T$). Pseudo labels were then paired with their corresponding images to create pseudo labelled data for training of ${\mathscr {M}}_S$. Initially, these models were trained using images having a size of $300 \times 300$. However, we observed that in images of this size, the size of the caries region is on average $10 \times 10$ and due to pixel imbalance in high-dimensional radiographs, the performance of self-supervised learning was not up to the mark. Therefore, to overcome this issue, we propose the use of centroid cropping for training ${\mathscr {M}}_T$ and ${\mathscr {M}}_S$ in a self-supervised learning strategy. Specifically, this approach works by cropping the caries region in high-dimensional radiographs and their corresponding label images. ${\mathscr {M}}_T$ was trained using cropped labelled training set and ${\mathscr {M}}_S$ was trained using cropped unlabelled images and their corresponding pseudo labels generated by ${\mathscr {M}}_T$. We used transfer learning, where the models were initially pre-trained on Microsoft’s COCO dataset^31^. All models were trained using a batch size of 8 with a learning rate of $10^{-3}$ for maximum epochs of 100. Furthermore, to prevent overfitting, we relied upon early stopping, which was based on the loss of five consecutive epochs.

### Performance evaluation

We have evaluated the performance of models trained using benchmark methods and our proposed efficient self-supervised learning approach using three widely used metrics: average pixel accuracy, mean intersection over union (mIoU), and dice score.

*Average pixel accuracy* is defined as the percentage of correctly classified pixels in the generated image (segmentation mask) from the model as defined below.

2 $$\begin{aligned} \text {mPA} = \frac{1}{k} \Sigma _{j=1}^k \frac{n_{jj}}{t_j}, \end{aligned}$$

where, mPA is the mean average pixel accuracy; $n_{jj}$ represents the total number of pixels that are correctly classified as label *j*, i.e., predicted and actual labels are the same (true positives); and $t_j$ is the total number of pixels that are classified as class *j*.

*Intersection over Union* (IoU) is a metric that is used to measure the overlap between two regions. In our case, IoU is used to quantify the overlap between the ground truth segmentation mask (labelled by an expert radiologist) and the segmentation mask predicted by our proposed method. Mathematically, it is computed as follows.

3 $$\begin{aligned} \text {IoU} = \frac{TP}{(TP+FP+TN)}, \end{aligned}$$

where, *TP* represents true positive, *FP* represents false positive, and *TN* represents true negative. Note that for segmentation problems, IoU is calculated using pixel-by-pixel analysis. The IoU can also be calculated as

4 $$\begin{aligned} \text {IoU}(X,Y) = \frac{|X \cap Y|}{|X \cup Y|}, \end{aligned}$$

*Dice similarity* is a widely used metric for evaluating the quality of segmentation in medical imaging. The dice score for a binary case (i.e., foreground and background segmentation) is calculated as:

5 $$\begin{aligned} \text {Dice\;score} = \frac{2TP}{2TP+FP+FN}, \end{aligned}$$

### Benchmark results for models trained using supervised learning

As discussed previously, to benchmark our dataset (DCD$^{2}$), we have evaluated six different state-of-the-art DL-based segmentation models for the tasks of caries segmentation using dental X-rays that include: (1) Deeplabv3-mobilenetv3; (2) Deeplabv3-resnet50; (3) Deeplabv3-resnet101; (4) FCN-resnet50; (5) FCN-resnet101; and (6) LRASPP-mobilenet-v3. All these models were trained in a supervised learning fashion using 90% of the data, and the remaining 10% was used for the evaluation. The results of these models in terms of three performance metrics are summarised in Table 1. The table highlights that the Deeplabv3 model with ResNet101 backbone outperformed all other models in terms of mPA, mIoU, and dice score. The remarkable performance of Deeplabv3 with ResNet101 backbone is mainly attributed to the architecture of ResNet101, as it has a comparatively much larger network with skip connections that enable efficient learning during training. Whereas, LRASPP-Mobilenet-v3 has provided the lowest performance, which is expected as it has a smaller architecture as compared to other models.

**Table 1:** Baseline results of six different models using fully supervised learning strategy.

Model	Backbone	Avg. accuracy	mIoU	Dice
Deeplabv3	ResNet-50	96.37	48.99	0.48
	**ResNet-101**	**98.38**	**50.18**	**0.50**
	Mobilenet-v3	96.10	48.05	0.48
FCN	ResNet-50	96.33	48.19	0.48
	ResNet-101	96.38	47.83	0.47
LRASPP	Mobilenet-v3	93.54	45.65	0.45

Significant values are in bold.

**Figure 5:**
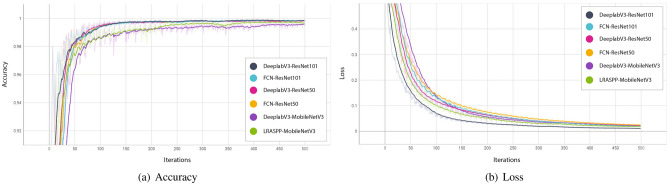
Models trained using the proposed self-training method demonstrate smooth learning behaviour in terms of accuracy and loss with an increase in iterations.

### Baseline results using student–teacher method-based self-training

**Figure 6:**
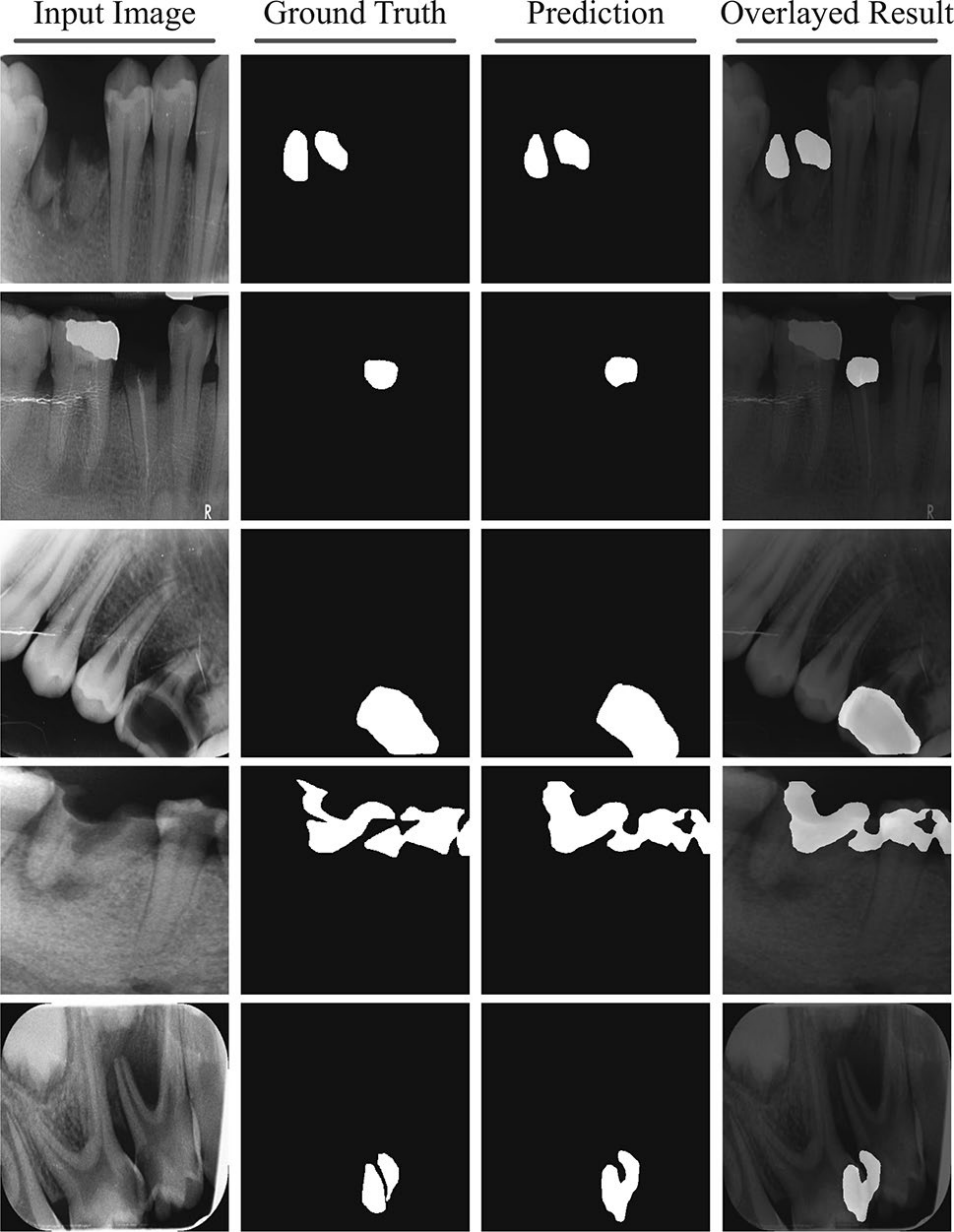
Qualitative results of proposed self-supervised learning strategy for caries detection in dental radiographs.

Over the last few years, utilizing unlabelled data along with the labelled dataset to train DL models in a semi-supervised fashion has received widespread adoption from the ML research community. Semi-supervised training-based methods such as self-training have been shown to be quite successful in leveraging unlabelled data and have provided competitive results as compared to fully supervised learning methods^32^. In this section, we present the baseline results for student-teacher method-based self-training when evaluated on DCD$^{2}$. Inspired by the supervised learning results, we selected the Deeplabv3 with ResNet101 backbone as a teacher model ${\mathscr {M}}_T$ (as it provided higher performance as compared to the other five models (Table 1)) and the remaining five models are trained as a student model ${\mathscr {M}}_S$ using self-training paradigm. To improve the performance of ${\mathscr {M}}_T$ and to ensure the efficacy of generated pseudo labels, the teacher model is trained using augmented data, i.e., high-dimensional images having a size of $300 \times 300$ and centrally cropped images having a size of $10 \times 10$ (as shown in Fig. 3). We randomly selected 20 images from the labelled training set for training of ${\mathscr {M}}_T$ and the remaining (labelled) images were considered as unlabelled images (i.e., the labels were ignored) that were used for the generation of pseudo labels (from trained ${\mathscr {M}}_T$ using 20 images). Whereas, the ignored ground truth labels were used to evaluate the efficacy of ${\mathscr {M}}_T$ in generating pseudo labels (visual examples depicting pseudo labels can be seen in Fig. 4). We used only real (human-labelled) test images to evaluate baseline models trained using self-supervised learning to ensure the effectiveness of the proposed method. The illustration of the learning behaviour of different models in terms of accuracy (Fig. 5a) and loss (Fig. 5b) is presented in Fig. 5. It is evident from the figure that models smoothly converge using our proposed CCS-based self-training approach. Furthermore, the results of these two approaches are summarised in Table 2. It is evident from the table that our proposed CCS-based self-training approach significantly outperformed the baseline self-training method in terms of all performance metrics. Also, we can see a similar trend as noted in fully supervised learning results, i.e., Deeplabv3 with ResNet101 backbone is providing superior performance as compared to other models. A visual depiction of model performance trained using our proposed method is presented in Fig. 6.

**Table 2:** Comparative analysis of different models trained using our proposed CCS-based self-training technique with standard self-training in terms of average accuracy (Avg. Acc), mean intersection over union (mIoU), and dice similarity score.

Standard self-training						CCS-based self-training					
Teacher model	Student model	Backbone	Avg. Acc	mIoU	Dice	Teacher model	Student model	Backbone	Avg. Acc	mIoU	Dice
Deeplabv3 (ResNet-101)	Deeplabv3	ResNet-50	91.77	45.07	0.45	Deeplabv3 (ResNet-101)	Deeplabv3	ResNet-50	94.57	46.35	0.46
		ResNet-101	93.22	47.83	0.47			**ResNet-101**	**99.43**	**50.73**	**0.50**
		Mobilenet-v3	90.15	44.38	0.44			Mobilenet-v3	95.08	45.60	0.45
	FCN	ResNet-50	90.04	44.81	0.44		FCN	ResNet-50	97.61	47.52	0.47
		ResNet-101	92.98	45.95	0.45			ResNet-101	95.84	45.87	0.45
	LRASPP	Mobilenet-v3	87.41	42.73	0.42		LRASPP	Mobilenet-v3	91.72	40.16	0.40

Significant values are in bold.

### Evaluating the effect of labelled data on teacher model for caries detection

We quantitatively evaluate the performance of teacher model training in the self-training paradigm by varying the number of labelled data samples. Specifically, we used 20, 40, 60, 80, and 120 real labelled images for training ${\mathscr {M}}_T$. The model is then evaluated using unlabelled data. In addition to the models that were evaluated using fully supervised learning (using real labelled data) and student-teacher method-based self-training. We evaluated three more state-of-the-art models in self-training strategy to demonstrate their efficacy on our DCD$^{2}$ that include PSPNet^33^, FPN^34^, and LinkNet^35^. These models are widely used for the evaluation and benchmarking of segmentation datasets. The quantitative results demonstrating the effect of a varying number of labelled samples on the performance of various models in terms of mIoU are summarized in Table 3. It can be seen from the table that all models provided superior performance when trained using 40 labelled images and their performance is least on 20 labelled images-based training. Moreover, we see that if we increase the number of labelled samples for training, the models start showing the overfitting behaviour, as their performance deteriorates with the increase of input samples (e.g., all models provided less performance when they were trained using 120 labelled images).

**Table 3:** Performance evaluation of various segmentation models by training on a different number of randomly sampled sets selected from the actual training set of our dataset for supervised learning.

Model	Backbone	No. of Labelled Samples				
		20	40	60	80	120
		mIoU				
FCN	ResNet-101	44.13	48.87	48.16	47.91	47.83
PSPNet	ResNet-101	42.84	45.91	45.29	44.50	44.28
LRASPP	MobileNet-v3	43.47	47.38	46.27	45.86	45.65
FPN	ResNet-101	44.21	48.65	47.95	47.33	47.06
LinkNet	ResNet-101	43.46	47.87	47.54	47.02	46.87
**Deeplab-v3**	**ResNet-101**	46.57	**52.41**	51.63	50.47	50.18

Significant values are in bold.

### Evaluating the effect of unlabeled data on student model for caries detection

In addition to evaluating the effect of varying labelled data on the performance of ${\mathscr {M}}_T$ in self-training, we also validated the performance of ${\mathscr {M}}_S$ by varying the number of unlabeled images (i.e., pseudo labelled samples). Moreover, we also analyze the effectiveness of our proposed CCS-based data sampling when ${\mathscr {M}}_S$ is trained with a different number of unlabelled images. Note that for these experiments we used Deeplabv3 with ResNet101 backbone, as these models provided superior performance as compared to other models. Also, the teacher model was trained using 40 images, as we got the best performance using this setting. Then we evaluated the performance of ${\mathscr {M}}_S$ by varying the number of pseudo-labelled samples, i.e., 10, 20, 40, 60, and 80. The quantitative results depicting the effect of varying pseudo-labelled samples on the performance of ${\mathscr {M}}_S$ trained using self-training strategy in terms of mIoU are presented in Table 4. We also present the results of ${\mathscr {M}}_S$ with and without CCS-based self-training to demonstrate the efficacy of our proposed data sampling technique. From Table 4, it is evident that the performance ${\mathscr {M}}_S$ being trained using self-supervised strategy increases with the increasing number of unlabelled (pseudo labelled) input samples. Moreover, we can see that our proposed CCS-based data sampling provides significant performance improvement in the training teacher models and as well as student models using self-training. The key reason behind the efficacy of CCS is the elimination of class-wise pixel imbalance in efficiently cropped images. This class imbalance issue arises due to class-wise pixel ratio, i.e., pixels belonging to the foreground (caries) and background in the high-dimensional images (where the foreground pixels are much smaller than the background pixels).

**Table 4:** Comparative analysis of student-teacher method-based self-training with and without proposed CCS-based data sampling.

Model	Real	Pseudo	w/o CCS	CCS
Teacher	40	–	52.41	56.49
Student	40	10	52.94	56.61
Student	40	20	54.73	58.34
Student	40	40	56.47	58.87
Student	40	60	56.98	59.42
Student	40	80	57.12	**59.76**

Significant values are in bold.

**Table 5:** Generalizability of student methods irrespective of different backbone network architectures on our dataset.

Model	Backbone	Val mIoU	Test mIoU
BiSeNet	ResNet-50	54.21	54.63
BiSeNet w/ CCS	ResNet-50	55.80	55.97
PSPNet	ResNet-101	53.42	53.66
PSPNet w/ CCS	ResNet-101	**56.18**	**56.43**
LRASPP	MobileNet-v3	49.74	49.86
LRASPP w/ CCS	MobileNet-v3	49.95	49.98
LinkNet	ResNet-101	53.61	53.85
LinkNet w/ CCS	ResNet-101	54.26	54.52

Significant values are in bold.

### Evaluating the generalization to different student models

In our all previous experiments, we used the same model architecture in the teacher and student models. Here we evaluate the generalizability of the self-training method across different architectures of student models using DCD$^{2}$ with and without our proposed CCS-based. Note that we used the same model (i.e., Deeplabv3-ResNet101) as the teacher model (owing to its superior performance in generating the pseudo labels). We used four different model architectures as the student model (including BiSeNet^36^, PSPNet, LRASPP, and LinkNet) and evaluated their performance of caries segmentation using validation data (taken from real labelled samples) and test data (unlabelled samples). Student model generalizability results are presented in Table 5, from the table is clear that our proposed self-training technique is generalizable across different student architectures as well. We see that the PSPNet model with ResNet-101 backbone outperformed all other models when trained using our proposed CCS-based data sampling technique. Also, it can be seen that the difference between the models’ performance on validation and test data is negligible that also demonstrates the effectiveness of pseudo labels generated by the teacher model.

## Conclusions

To address the problem of data scarcity and the reduced cost associated with annotation in medical imaging, we present a student-teacher method-based self-supervised learning approach for dental caries detection that uses both labelled images and unlabelled images. We first present a dental X-ray image database, which is annotated by a team of experts trained by an expert dental radiologist (having experience of more than 20 years). Then, we present a centroid cropping-based approach for dynamically cropping the caries region in dental X-ray images, which is used for the training of models in a self-supervised learning fashion. Centroid-cropped images have much smaller dimensions as compared to original (high-dimensional) images and have also outperformed models trained using original data in self-supervised learning settings. Our method works by only utilising 20 labelled images, and the rest of the images are considered unlabelled for training models in self-supervised learning (we got best results when 40 labelled images are used). We have compared our proposed approach with a baseline fully supervised learning strategy (in which models are trained with fully labelled data) and self-supervised learning (where the models are trained using high-dimensional images). Also, we perform an extensive evaluation of the proposed method to ensure better generalizability. Our experiments demonstrate that our approach outperformed baseline methods in terms of average pixel accuracy, mean intersection over union (mIoU), and dice score. Our future work includes the development of a more diverse and larger database for dental caries detection.

## Data Availability

The code along with data collected and analysed in this study is available at this GitHub repository (https://github.com/madnanq/dental-caries-detection). The data collected and analysed in this paper is available from the corresponding author upon reasonable request.
